# Refining Methodological Approaches in Pediatric Neurosurgery: Considerations for Future Research

**DOI:** 10.1590/S1677-5538.IBJU.2025.0167

**Published:** 2025-04-20

**Authors:** Shengyi Chen, Yuekun Fang, Bin Cheng

**Affiliations:** 1 Wenzhou Hospital of Integrated Traditional Chinese and Western Medicine Department of Andrology Wenzhou China Department of Andrology, Wenzhou Hospital of Integrated Traditional Chinese and Western Medicine, Wenzhou, China; 2 Wenzhou Hospital of Integrated Traditional Chinese and Western Medicine Department of Urology Wenzhou China Department of Urology, Wenzhou Hospital of Integrated Traditional Chinese and Western Medicine, Wenzhou, China

To the editor,

We are writing to express our appreciation for the recently published study titled "The Effect of Detethering Surgery on the Bladder Function and Psychology of Children with Primary Tethered Cord Syndrome" in the International Braz J Urol (1). This study provides valuable insights into the impact of detethering surgery (DS) on bladder function and psychological behavior in pediatric tethered cord syndrome (TCS) patients. The authors’ meticulous approach to evaluating postoperative outcomes has significantly contributed to the understanding of neurogenic bladder management and post-surgical recovery. In particular, the study's emphasis on both functional and psychological parameters offers a comprehensive perspective on the multifaceted nature of TCS management. Despite the study's commendable contributions, we would like to offer constructive suggestions for future improvements.

First, the choice of statistical models warrants further refinement. The study employs standard comparative analyses to evaluate bladder function outcomes; however, incorporating cluster analysis could provide additional insights (2). By grouping patients based on preoperative bladder characteristics or surgical response patterns, researchers could better identify subgroups that benefit most from DS. This stratified approach would enhance the study's applicability and clinical relevance.

Second, potential confounding variables merit additional consideration. While the study adjusts for age, gender, and baseline bladder function, other factors—such as socioeconomic status, prior interventions (e.g., pharmacological treatments or catheterization practices), and the severity of spinal cord tethering—may significantly impact postoperative outcomes. Incorporating these variables into the analytical framework would strengthen the study's robustness and improve result interpretation.

Third, the duration and sustainability of treatment effects warrant further exploration. The study assesses bladder function within a relatively short follow-up period. However, the long-term progression of neurogenic bladder dysfunction in TCS patients remains uncertain. Future studies should extend the follow-up duration to evaluate whether initial improvements persist over time or if secondary complications arise. Additionally, standardized postoperative management strategies should be considered to ensure consistent patient outcomes.

In conclusion, this study provides compelling evidence on the effects of DS in pediatric TCS patients, particularly regarding bladder function and psychological outcomes. However, refining statistical methods, accounting for additional confounding factors, and extending follow-up periods could further enhance the study's impact. We commend the authors for their valuable contribution and look forward to continued advancements in this field.

The Authors
